# Inhibition of Pyruvate Kinase From *Thermoanaerobacterium saccharolyticum* by IMP Is Independent of the Extra-C Domain

**DOI:** 10.3389/fmicb.2021.628308

**Published:** 2021-02-17

**Authors:** Christopher A. Fenton, Qingling Tang, Daniel G. Olson, Marybeth I. Maloney, Jeffrey L. Bose, Lee R. Lynd, Aron W. Fenton

**Affiliations:** ^1^Department of Biochemistry and Molecular Biology, The University of Kansas Medical Center, Kansas City, KS, United States; ^2^Thayer School of Engineering, Dartmouth College, Hanover, NH, United States; ^3^Oak Ridge National Laboratories, Center for Bioenergy Innovation, Oak Ridge, TN, United States; ^4^Department of Microbiology, Molecular Genetics and Immunology, The University of Kansas Medical Center, Kansas City, KS, United States

**Keywords:** allosteric regulation, allostery, pyruvate kinase, ethanol, metabolic engineering

## Abstract

The pyruvate kinase (PYK) isozyme from *Thermoanaerobacterium saccharolyticum* (TsPYK) has previously been used in metabolic engineering for improved ethanol production. This isozyme belongs to a subclass of PYK isozymes that include an extra C-domain. Like other isozymes that include this extra C-domain, we found that TsPYK is activated by AMP and ribose-5-phosphate (R5P). Our use of sugar-phosphate analogs generated a surprising result in that IMP and GMP are allosteric inhibitors (rather than activators) of TsPYK. We believe this to be the first report of any PYK isozyme being inhibited by IMP and GMP. A truncated protein that lacks the extra C-domain is also inhibited by IMP. A screen of several other bacterial PYK enzymes (include several that have the extra-C domain) indicates that the inhibition by IMP is specific to only a subset of those isozymes.

## Introduction

**FIGURE 1:**
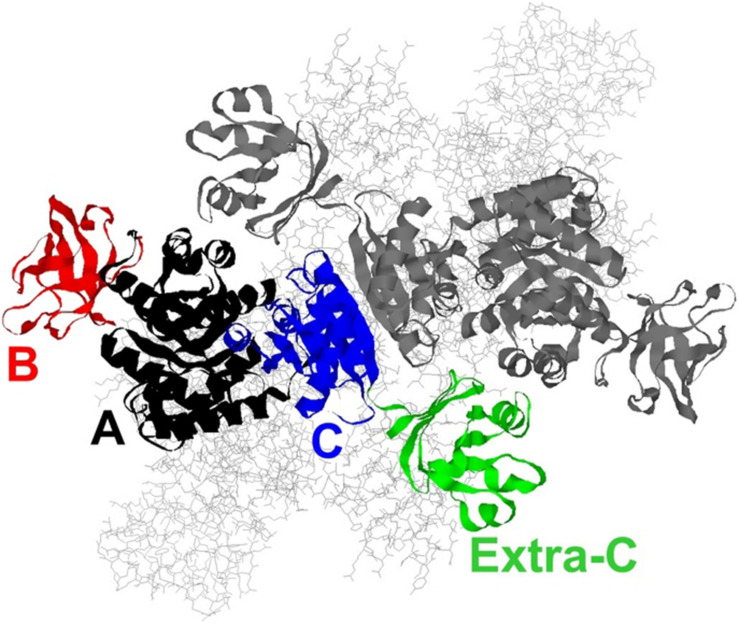
A model of the TsPYK homotetramer built by SwissModel (Biasini et al., 2014). The two subunits in the background are rendered as gray sticks. The two subunits in the foreground are rendered as ribbons: the top subunit is gray; the bottom is colored by domain. The A domain is in black, the B in red, the C in blue, and the extra-C in green.

*Thermoanaerobacterium saccharolyticum* is a thermophilic bacterium that has been engineered to produce ethanol from hemicellulose at high yield (∼90% of theoretical) and titer (70 g/L), but is poor at using cellulose for that production (Herring et al., 2016). In contrast, *Clostridium thermocellum* has a strong native ability to consume cellulose, but its ability to produce ethanol is relatively modest (Olson et al., 2012, 2015). To address if the lack of pyruvate kinase expression in *C. thermocellum* is a limit in the cellulose-to-ethanol metabolism, heterologous expression of pyruvate kinase (PYK) from *T. saccharolyticum* (TsPYK) in *C. thermocellum* increased ethanol yield slightly (12% improvement). A further 21% improvement was realized when the competing “malate shunt” pathway was disrupted, forcing carbon flux through the PYK reaction (Deng et al., 2013; Olson et al., 2017). Given the success of this design, it is interesting to note that the regulatory features of TsPYK have not previously been studied and addressing that knowledge gap was the goal of the current study. The sequence of TsPYK places this enzyme in a subfamily of PYK isozymes that includes an extra-C domain (Munoz and Ponce, 2003). Munoz and Ponce (2003) identified strains of *Lactococcus*, *Lactobacillus* and *Bacillus* with the extra-C domain. Other organisms whose PYK enzyme contains the extra-C domain include the mesophilic organisms *Clostridium cellulolyticum*, *Staphylococcus aureus* and *Synechocystis* sp. PCC6803 and the thermophilic organisms *T. saccharolyticum*, *Thermoanaerobacterium thermosaccharolyticum*, *Caldicellulosiruptor bescii*, and *Clostridium clariflavum*. This domain in the “extra-C domain” subfamily is in addition to the three domains (A, B, and C) common to all known PYK isozymes (Figure 1).

A function for the extra-C domain has yet to be elucidated, however, deletion studies have been performed in two organisms: *Geobacillus stearothermophilus* and *S. aureus*. When this domain was removed from the *G. stearothermophilus* PYK, the affinities for PEP and ribose-5-phosphate (R5P; the allosteric effector) were decreased, as was thermostability (Sakai, 2004). In *S. aureus*, deletion of the extra-C domain decreased enzyme efficiency (*k*_*cat*_/*S*_0.5_) toward PEP and ADP to about 5% of the wild type level (Zoraghi et al., 2010).

In this study, our efforts to characterize the regulation of TsPYK combine two approaches: (1) the use of sugar-phosphate analogs was included to evaluate which chemical moieties of the effector are required to elicit allosteric activation (i.e., increased binding of the substrate phosphoenolpyruvate, PEP) and (2) the allosteric functions were evaluated in TsPYK truncated to remove the extra-C domain to determine if allosteric regulations are dependent on that extra-C domain. Activation by R5P and AMP are similar suggesting that it is the ribose moiety of AMP that serves as the allosteric activator. However, this approach identified the first example of a PYK isozyme that is inhibited by IMP and GMP. IMP inhibition is specific to only a subset of isozymes surveyed.

## Materials and Methods

### Protein Purification

A 6XHis-tag was added on the N-terminus of TsPYK in the pTrcHisb plasmid and the tagged protein was expressed in *E. coli* FF50 cells (in which both native *E. coli pyk* genes have been deleted) (Fenton and Hutchinson, 2009) using the ampicillin selection marker. Frozen cell pellets were resuspended in 50 mM HEPES pH 7.2, 5 mM MgCl_2_, 300 mM KCl, 10 mM imidazole, and 10 mM TCEP (added fresh). Samples were sonicated on ice water using the same on/off pulse sequence. Sonication included 5 s “on” pulses separated by 45 s “off” pulses for a total “on” time of 4 min. The insoluble fraction was removed *via* centrifugation. Samples were incubated at 55°C for 1 h (Supplementary Material) and the insoluble fraction, including heat precipitated proteins, was removed *via* centrifugation. The supernatant was added to an agarose Ni^2+^ column (Biorad Profinity Ni^2+^ resin). The column was washed with two bed volumes of the HEPES buffer (the same as used for sonication) and the TsPYK protein was eluted with a gradient of 10–500 mM imidazole in the same HEPES-based buffer. Fractions with PYK activity were pooled and the pool was dialyzed into the sonication buffer with 10 mM imidazole. The 6XHis tag was not removed; we acknowledge that the purification tag can alter the magnitude of allosteric responses, but the regulatory features characterized with the tagged protein are likely to be qualitatively present on the untagged protein. Purified TsPYK were frozen *via* aliquoting small quantities into thin walled PCR tubes and plunge freezing into liquid nitrogen (Deng et al., 2004). Long term storage was at −80°C (activity recovered over 2 months as tested here).

Over the course of the study, we identified the need for higher levels of protein expression and more consistent expression, both of which can be gained by using BL21 *E. coli* expression systems. To improve expression levels and the consistency of expression, a new strain of *E. coli* was generated from the parent BL21 strain. The two deletion cassettes used in the construction of FF50 (Kan^*R*^ gene flanked by homology regions for the respective gene and recognition sites for the FLP recombinase (FRT) (Fenton and Hutchinson, 2009) were transferred to BL21 (DE3) using P1 lysates (Miller, 1992). After each cassette transfer, the kanamycin selection marker was removed using a temperature-sensitive plasmid-encoded FLP recombinase (pCP20) as previously reported (Cherepanov and Wackernagel, 1995; Lovingshimer et al., 2006). Deletion of the two genes caused loss of PYK activity as expected (Supplementary Material). To express the TsPYK protein in the newly created BL21 *E. coli* strain, now named QTF60, TsPYK gene was cloned with an N-terminal tag consisting of 6XHis-MBP-SUMO- (MBP: maltose binding protein) in the pCDF (Novagen) expression plasmid and transformed into QTF60. Purification was initiated with a frozen pellet harvested from a 1.5 L cultures grown with 50μg/ml spectinomycin and induced with 1.8 g/L lactose. The 6XHis-MBP-SUMO-TsPYK protein was purified very similar to that presented above for the 6XHis-TsPYK protein. The buffer used for sonication included 50 mM imidazole instead of the 10 mM listed above. After the centrifugation step of the 55°C-treated sample, the supernatant from that sample was ammonium sulfate (AS) fractionated with two sequential additions of AS. The first addition was 0.2 g AS/ml sample, followed by centrifugation. To the supernatant, an additional 0.12 g AS/ml sample was added, followed by centrifugation. The pelleted protein was resuspended in a small volume (∼0.5 ml) of sonication buffer. The dialyzed sample was then purified with a Ni^2+^ column using a 50–500 mM imidazole gradient to elute protein. Purified TsPYK were frozen *via* plunge freezing. Long term storage was at −80°C. Other bacterial PYK proteins included in the screen were also cloned with the 6XHis-MBP-SUMO- tag in pCDF, expressed in the QTF60 cells, and purified and stored as described for TsPYK (with the exception that the heat step and ammonium sulfate steps were excluded).

### Kinetic Assays and Data Analysis

All methods used for collecting initial velocity data and to evaluate allosteric properties are identical to those used to in the characterization of human LPYK (Ishwar et al., 2015), with the exception of Mg^2+^, ADP, and final K^+^ and Na^+^ concentrations. Briefly, activity measurements were performed at 30°C, in an assay containing 50 mM HEPES, 15 mM MgCl_2_, 10 mM (K)ADP, 0.1 mM EDTA, 0.18 mM NADH, 10 mM DTT, and 19.6 U/mL lactate dehydrogenase. PEP and effector concentrations were varied. The rate of NADH utilization was monitored at A_340_ for each concentration of PEP and these initial velocity rates as a function of PEP concentration were used to evaluate *K*_*app*–*PEP*_ at any one effector concentration. In turn, *K*_*app*–*PEP*_ evaluated over a concentration range of effector was used to evaluate allosteric parameters. To prevent a large percent change in K^+^ or Na^+^ concentrations, KCl and NaCl were added for final concentrations of 195 mM K^+^ and 80 mM Na^+^ (Fenton and Alontaga, 2009).

*K*_*app*–*PEP*_ values were obtained by fitting initial rates obtained from kinetic assay to:

(1) $$v=\frac{V_{m\,a\,x}\,{\left[P\,E\,P\right]}^{n_{H}}}{{\left(K_{a\,p\,p-P\,E\,P}\right)}^{n_{H}}+{\left[P\,E\,P\right]}^{n_{H}}},$$

where *V*_*max*_ is the maximum velocity, *K*_*app*–*PEP*_ is the concentration of substrate that yields a rate equal to one-half the *V*_*max*_, and n_*H*_ is the Hill coefficient. The allosteric coupling constant is defined as (Reinhart, 2004):

*Q*_*ax*_ = *K*_*ia*_/*K*_*ia/x*_ = *K*_*ix*_/*K*_*ix/a.*_ (2)

*Q*_*ax*_ was determined by fitting a plot of the *K*_*app*–*PEP*_ values as a function of effector concentration to Eq. 3 (Reinhart, 2004):

(2) $$K_{\mathrm{app}-\mathrm{PEP}}=K_{a}\,\left(\frac{K_{\text{ix}}+\left[\text{Effector}\right]}{K_{\text{ix}}+Q_{\text{ax}}\,\left[\text{Effector}\right]}\right)$$

where *K*_*a*_ = *K*_*app*–*PEP*_ when [Effector] = 0; *K*_*ix*_ = the dissociation constant for effector (X) binding to the protein in the absence of substrate (A). For this second level of data fitting, error estimates from the initial fit to Eq. 1 were used as weighting factors in the fit to Eq. 3, thus error was propagated to the final fit values. An aid for visualizing fit parameters included in Eq. 3 in the figures can be found in Supplementary Material.

## Results and Discussion

Consistent with other PYK isozymes, the allosteric response of TsPYK was a change in the apparent affinity of the enzyme for the PEP substrate with no change in *V*_*max*_ activity. Throughout the PYK family, ADP affinity is also not responsive to effectors. Because the allosteric response causes a change in PEP affinity, all results are shown as the response of the *K_*app*–*PEP*_* value (i.e., the kinetic derived “affinity” value) over the concentration range of effector evaluated. Fit parameters are included in Table 1.

**TABLE 1:** Fit parameters.

***K_*app*–*PEP*_*** [mM]	0.630.03		
***K_*ix*–*AMP*_*** [mM]	0.090.06	***Q_*ax*–*AMP*_***	2.00.2
***K_*ix*–*R*5P_*** [mM]	1110	***Q_*ax*–*AMP*_***	3.00.7
***K_*ix*–*GMP*_*** [mM]	11	***Q_*ax*–*GMP*_***	0.260.09
***K_*ix*–*IMP*_*** [mM]	31	***Q_*ax*–*IMP*_***	0.290.04

**FIGURE 2:**
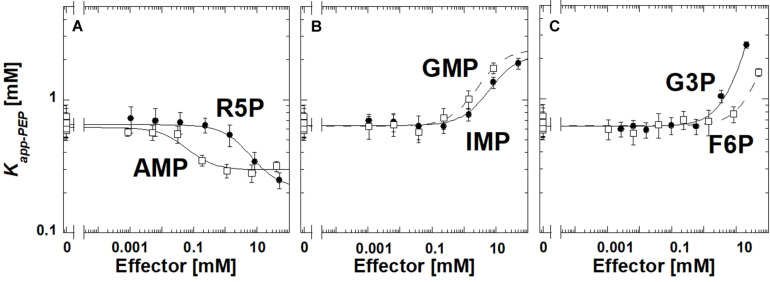
The allosteric response of TsPYK to various effectors **(A–C)**. R5P is ribose-5-phosphate. F6P is fructose-6-phosphate. G3P is glyceraldehyde 3-phosphate. Error bars represent the estimated parameter error provided by the fitting program from the fitting of the primary data to Eq. 1.

Ribose-5-phosphate is also known to activate several PYK isozymes (Wilke and Schlegel, 1975; Mort and Sanwal, 1978; Mertens et al., 1992; Le Bras and Garel, 1993; Valentini et al., 1993; Suzuki et al., 2008; Snasel and Pichova, 2019). AMP includes a ribose-5-phosphate moiety in its total structure. Furthermore, ribose-5-phosphate shares the ribose ring with the fructose-1,6-bisphosphate (Fru-1,6-BP) activator of mammalian PYK isozymes (Hall and Cottam, 1978; Blair, 1980; Ishwar et al., 2015). Considered together, one possibility is that AMP regulates TsPYK *via* the ribose-5-phosphate interacting with the site on TsPYK that is equivalent to the Fru-1,6-BP binding site of mammalian PYK isozymes. Consistent with that idea, ribose-5-phosphate elicits an allosteric response (in Figure 2, the difference between the plateaus at low and high effector concentrations) that is very similar to that caused by AMP. If these two effectors bind to the same site on the protein, then it seems reasonable to interpret that the adenine ring of AMP must contribute additional interactions that increased effector affinity (Figure 2). The binding site of these effectors is believed to be equivalent to the location at which Fru-1,6-BP binds in mammalian PYK isozymes (Jurica et al., 1998; Valentini et al., 2002; Holyoak et al., 2013).

To initiate a study to identify the minimum chemical substructure from AMP/ribose-5-phosphate effector that is required for allosteric regulation, we employed additional analogs. 2-deoxyadenonsine-5′MP generated a response very similar to AMP (slightly reduced binding), therefore, the 2′hydroxide contributes little to the allosteric mechanism (Supplementary Material). *K_*app*–*PEP*_* did not change upon the addition of Fru-1,6-BP, 3-phosphoglycerate or ribose (data not shown); the lack of response does not distinguish between a ligand failing to bind or binding without eliciting an allosteric response.

Interestingly, glyceraldehyde 3-phosphate, fructose-6-phosphate, IMP or GMP all caused reduced affinity for PEP, rather than increasing that affinity (Figure 2). Other phosphorylated 3-carbon sugars (e.g., 3-phosphoglycerate and glyceraldehyde-3-phosphate) have already been shown to regulate PYK isozymes (Solomons et al., 2013; Ishwar et al., 2015), but the outcomes are routinely increased PEP affinity, rather than the reduced PEP affinity identified in this study. Also, ribose-5-phosphate has also been listed as an inhibitor of at least one PYK isozyme (Lin et al., 1989). In this study, within the working concentrations of IMP (up to 50 mM), the shift in *K_*app*–*PEP*_* reaches a plateau, indicating saturation of the effector binding site. However, a plateau at high concentrations of GMP, fructose-6-phosphate, or glyceraldhehyde-3-phosphate was not apparent.

The newly discovered inhibition by IMP and GMP was particularly interesting to us. Therefore, we next addressed if the extra-C domain might be responsible for this previously uncharacterized type of regulation. We also questioned if the newly discovered inhibition was common in the subfamily of proteins that include the extra-C domain and/or other PYK isozymes from microbe sources. To address these two questions using purified protein, we designed an expression system that added a 6XHis-MPB-SUMO- tag onto the N-terminus of each protein screened. To improve protein expression, this new expression design was included in a pET vector and expressed in a BL21 (DE3) cell line in which the two *E. coli* PYK genes have been deleted (QTF60, developed in this study, see Supplementary Material). The screen of the influence of IMP on various PYK isozymes was completed with the 6XHis-MBP-SUMO- tag remaining on the PYK enzymes. In addition to the TsPYK full length and TsPYK extra-C domain truncated proteins, the PYK isozymes included in the screen were *E. coli* Type F (Gene ID 946179; protein ID YP003054274.1) (Mattevi et al., 1996), *E. coli* Type A (Gene ID 946527; protein ID AAA24473.1) (Malcovati et al., 1973; Somani et al., 1977; Malcovati and Valentini, 1982), *C. bescii* (Gene ID 31772614; protein ID WP_015907751.1), *C. cellulolyticum* (protein ID WP_015925984.1), *C. clariflavum* (protein ID WP_014254383.1), *S. aureus* (Gene ID 59700435, protein ID WP_001232648.1) (Zoraghi et al., 2010), *T. thermosaccharolyticum* (protein ID ADL68325.1), *G. stearothermophilus* (Gene ID 58572631; protein ID BAA02406.1) (Sakai et al., 1986; Sakai and Ohta, 1987, 1993; Walker et al., 1992; Nguyen and Saier, 1995; Lovell et al., 1998; Sakai, 2004, 2005; Suzuki et al., 2008; Ueda and Sakasegawa, 2019), *Synechocystis* (protein ID BAA17574.1) (Knowles et al., 2001), and *L. delbrueckii* (Gene ID 57117943; protein ID CAA50527.1). All but the truncated TsPYK and the two *E. coli* enzymes include the extra-C domain.

**FIGURE 3:**
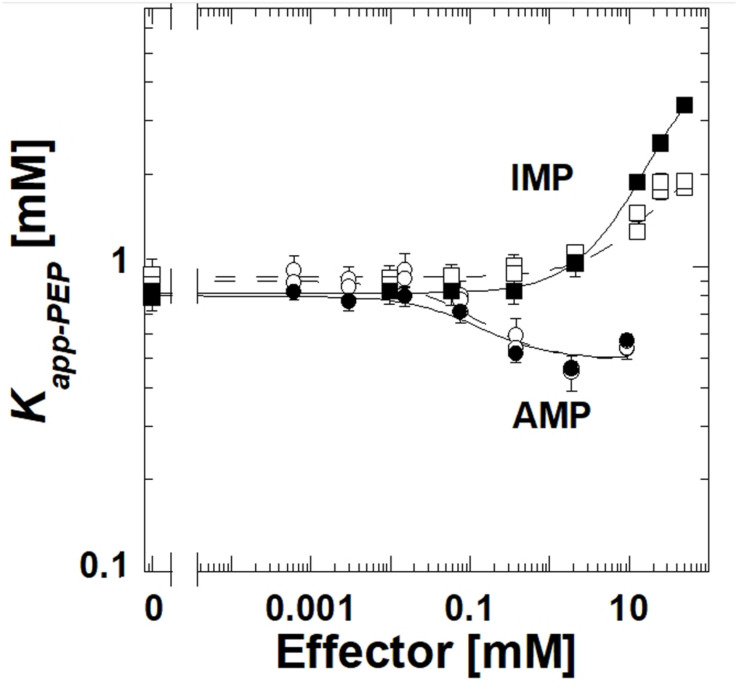
A comparison of responses to IMP and AMP for the full length TsPYK (filled symbols/solid lines) and the truncated form that lacks the extra-C domain (open symbols/dashed lines); both include the MBP tag. Responses to AMP are circles and to IMP are squares.

The deletion of the extra-C domain may influence the magnitude of the response to IMP, however, it is clear that when the extra-C domain was deleted from TsPYK, the truncated protein continued to be inhibited by IMP (Figure 3). Activation by AMP was not influenced by the removal of the extra-C domain.

**FIGURE 4:**
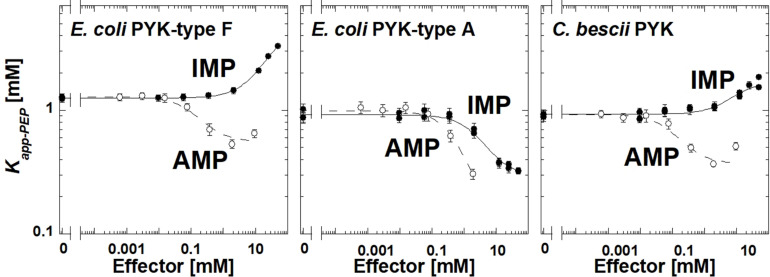
The response of three bacterial PYK isozymes to AMP and IMP. Additional data for other bacterial PYK isozymes are included in Supplementary Material.

The *E. coli* type F isozyme and the isozyme from *C. bescii* were inhibited by IMP and activated by AMP similar to TsPYK (Figure 4). However, the *E. coli* type A isozyme was activated by both AMP and IMP. The PYK isozymes from *C. cellulolyticum*, *C. cariflavum*, *G. stearothermophilus*, *S. aureus*, *Synechocystis*, and *T. thermosaccharolyticum* had little or no response to the concentration ranges of IMP and AMP used in this study (Supplementary Materials). Clearly, the response to IMP is not common across the microbial PYK isozymes included in this study and therefore, the response to that effector is not dependent on the extra-C domain.

To the best of our knowledge, this is the first report that IMP and GMP act as inhibitors of any PYK isozyme. Both *E. coli* Type F enzyme (which lacks the extra-C domain) and TsPYK with the extra-C domain truncated are inhibition by IMP. Therefore, the IMP inhibition does not appear to be a function of the extra-C domain. A more recently discovered second phosphate binding site (Zhong et al., 2017) is not conserved in these isozymes and is not likely a binding site for IMP. That leaves the possibility for the inhibiting IMP and the activating AMP to compete for binding to the same allosteric site. Certainly, there are other allosteric systems in which allosteric inhibitors and allosteric activators bind to the same binding site (Shirakihara and Evans, 1988; Zorba et al., 2019). In the case of these bacterial PYK examples, total PYK activity may be influenced by competitive binding of the inhibitor and activator to the same site, a scenario that would be very sensitive to changing cellular concentrations of these two effector ligands.

## Conclusion

In this work, we have demonstrated several important regulatory characteristics of the TsPYK enzyme, namely that it is activated by AMP and R5P and that it is inhibited by IMP and GMP. In our effort to consider how TsPYK contributes to metabolism when expressed in *C. thermocellum*, we can first point out that *C. thermocellum* has a number of glycolytic reactions with atypical cofactor specificity (Zhou et al., 2013). As a result, one possible explanation for the low levels of carbon flux through the TsPYK-catalyzed reaction in *C. thermocellum* is that TsPYK is inhibited due to high levels of GMP. As a contrasting example that extrapolates beyond the current study, it may be that cyclic-GMP is the relevant regulator: cyclic-GMP has been identified as a critical signaling molecule in some bacteria (Amikam and Benziman, 1989; Morgan et al., 2014). However, the possibility that GMP (or cyclic-GMP) inhibits TsPYK in the engineered *C. thermocellum* also depends on concentrations of other nucleotide monophosphates (e.g., AMP), that will bind to TsPYK competitively with GMP and activate instead of inhibiting the enzyme. Clearly, whether inhibition will result from the collective influence of GMP and IMP or activation will result from the collective influence of multiple activating nucleotide monophosphates will be determined by small changes in the concentration of each molecule. In addition, because AMP binds tighter than GMP, GMP concentrations must be higher than those of AMP for the GMP inhibition to have a physiological implication. Nonetheless, the inhibition by GMP remains a possible explanation for lower than expected influences of TsPYK on *C. thermocellum* metabolism. The reduced influence in the previous metabolic engineering studies may be less likely if thermophilic PYK enzymes that lack inhibition by GMP are expressed in *C. thermocellum*.

## Data Availability Statement

The raw data supporting the conclusions of this article will be made available by the authors, without undue reservation, to any qualified researcher.

## Author Contributions

CF, QT, and AF purified all proteins and collected kinetic responses. DO, MM, JB, and LL designed and cloned plasmid constructs. AF, DO, and JB contributed to the writing of this manuscript. All authors contributed to the article and approved the submitted version.

## Conflict of Interest

LL was a founder of the Enchi Corporation, which has a financial interest in *C. thermocellum*. The remaining authors declare that the research was conducted in the absence of any commercial or financial relationships that could be construed as a potential conflict of interest.
