# Supplementary material for: Inhibition of Pyruvate Kinase From Thermoanaerobacterium saccharolyticum by IMP Is Independent of the Extra-C Domain
Source: Front Microbiol. 2021 Feb 17;12:628308. doi: 10.3389/fmicb.2021.628308 (PMC7925390; doi:10.3389/fmicb.2021.628308)
Supplement: Supplementary file 1 [file Data_Sheet_1.docx]

Supplementary Material

**Partial protein purification of TsPYK without a purification tag**

For evaluations of partially purified TsPYK, the TsPYK gene was cloned into pTrcHisB expression plasmids and transformed into *E. coli* FF50 cells (in which both native *pyk* genes have been deleted) ([9](#_ENREF_9)) using the ampicillin selection marker. 1.5L LB cultures were grown with 0.10g/L ampicillin and 1.8g/L lactose to induce expression (added at inoculation). Cell pellets were harvested *via* centrifugation and frozen until use. Purification was influenced by conditions used to purify *E. coli* pyruvate kinase Type F out of *E. coli* ([26](#_ENREF_26)). Frozen cell pellets were suspended in purification buffer (10mM Tris pH 8.5, 100mM KCl, 1mM EDTA, and 2mM 2-mercaptoethanol) and sonicated. Sonication was completed while samples were surrounded by ice water. Samples were sonicated on ice water using the same on/off pulse sequence. Sonication included 5 second “on” pulses separated by 45 second “off” pulses for a total “on” time of 4 minutes. The insoluble fraction was removed *via* centrifugation and used to evaluate the concentration of ammonium sulfate required to precipitate this protein and the resistance to loss of activity at various temperatures (Supplemental Figure 1).

Supplemental Figure 1. Assays of crude extracts treated with different temperatures (A) or different ammonium sulfate concentrations (B). A) Heat treated samples were placed at the respective temperature (°C) for 1hr. Activity is reported as the percent activity remaining at the given temperature compared to the activity remaining after 1 hr. at 55°C. Temperature data in panel A represents the average and standard deviation of 8 replicates. (B) Indicated ammonium sulfate concentrations were added separate aliquots of supernatants after cell lysis. Samples were centrifuged and the PYK activity remaining in solution was evaluated. Activity is reported as the % remaining in solution compared to the activity in the absence of ammonium sulfate. Ammonium sulfate data in panel B represents the average and standard deviation of 5 replicates.

**Supplemental Figure 2.** A visual aid to help the reader identify parameters in other Figures. Binding constants for the effector are NOT equal to the midpoint of the response. Here we marked the two effector binding constants (*K_ix-IMP_* and *K_ix-AMP_*) as derived from the data fits.

**Influence from ions**

Early work in our study that used partially purified samples resulted in highly variable *K_app-PEP_* values. This variability was removed by more extensive dialysis after exposure to ammonium sulfate. We further explored the potential that residual ammonium sulfate influenced observed *K_app-PEP_* values by monitoring *K_app-PEP_* over concentration ranges of various salts. The *K_app-PEP_* of wild type TsPYK responds to sulfate containing salts at ~1mM, a low concentration relative to those used for ammonium sulfate fractionation. The *K_app-PEP_* value for TsPYK is even more sensitive to phosphate ions than sulfate ions.

Supplmental Figure 3. The influence of salt type and concentration on *K_app-PEP_* values. All salt concentrations listed are in addition to the 195mM K^+^ from ligands and buffer addtions (but without the 80mM Na^+^ added in other assays as reported in Materials and Methods).


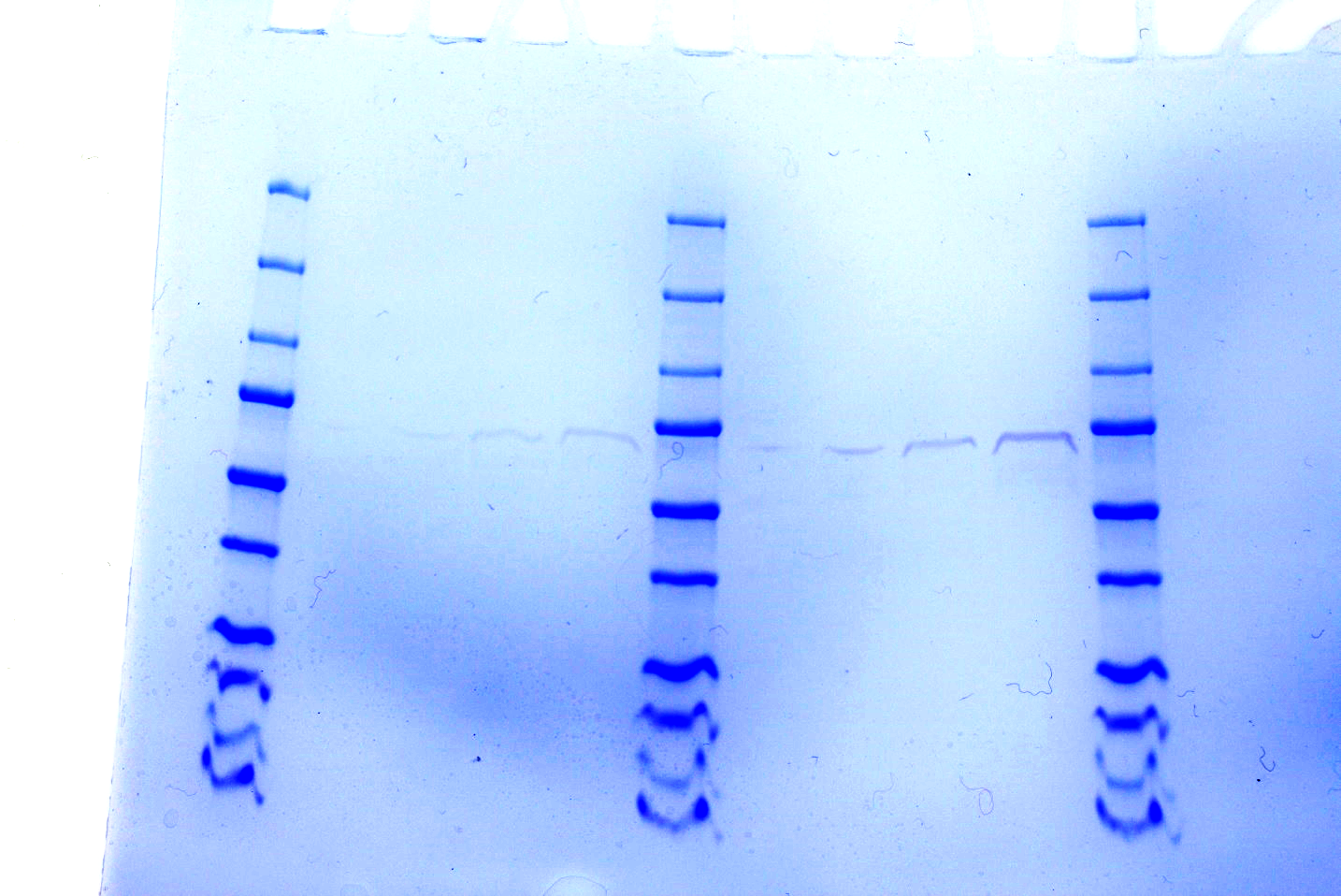


250kD

150kD

100kD

75kD

50kD

37kD

25kD

20kD

15kD

10kD

Supplemental Figure 4. Coomassie stained 7-15% gradient SDS-PAGE of standard (left) and Ni^2+^ column purified 6XHis-TsPYK (right).

Supplemental Figure 5. Comparative data for the response to AMP and to 2-deoxyadenonsine-5’MP.

BL21 (DE3)

BL21 (DE3)

ΔPYKA

BL21 (DE3)

ΔPYKF

QTF60

**Strain**

Supplemental Figure 6. Activity remaining after deleting genes from BL21 (DE3) *E. coli*. The two deletion cassettes used in the construction of FF50 were transferred to BL21 (DE3). As expected, the deletion of both *E. coli* PYK genes resulted in a loss of PYK activity in cellular extracts. Therefore, TsPYK and other PYK isozymes were expressed in this newly created BL21 *E. coli* strain, now named QTF60.

250kD


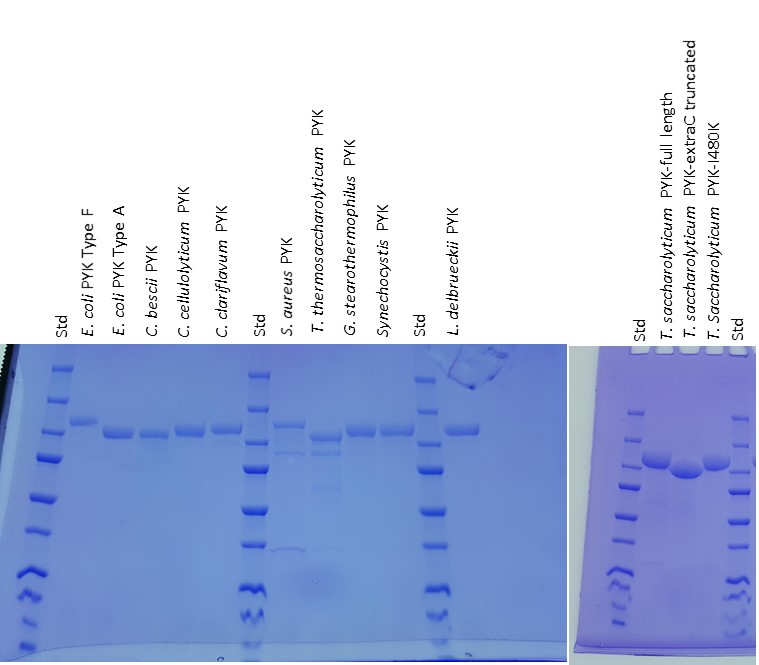


150kD

100kD

75kD

50kD

37kD

25kD

20kD

15kD

10kD

Supplemental Figure 7. Coomassie stained 4-15% gradient SDS-PAGE of Ni^2+^ column purified 6XHis-MBP-SUMO-proteins.

Supplemental Figure 8. Additional data (to that included in Figure 4) from the screen of PYK isozymes. Isozymes included here showed very little response to AMP or IMP. *G. stearothermophilus* PYK was previously reported to be activated by AMP (H. Sakai, K. Suzuki, and K. Imahori, Purification and properties of pyruvate kinase from *Bacillus stearothermophilus*, J. Biochem 99 (1986) 1157-1167.). Please note that *B. stearothermophilus* has now been renamed as *G. stearothermophilus*. Our data did not find that same activation for the tagged-protein. Therefore, we tested for consistency in our assay system using the PYK enzyme from “*Bacillus stearothermophilus*” from Sigma Chemical Co. (data below).

Therefore, these two samples should be equivalent with the exception of the N-terminal purification tag used in our expression system. Data for enzyme purchased from Sigma Chemical Co. in open squares. Data for in-house purified enzyme with N-terminal purification tag in solid circles. Data for the tagged protein serves as a replicate for the data for this enzyme above. Although data for both proteins lack a response to AMP, the work by Sakai *et al*. suggested that contamination of ADP by AMP could cause activation. The added purification of ADP to remove AMP contamination, as used by Sakai *et al*., was not included in our study and may be an explanation for the lack of an AMP response for the G_S_PYK protein. However, the conditions used here were designed to be comparative with the conditions that resulted in allosteric responses from TsPYK.
